# The Interaction of Complement and Intrinsic Coagulation System: A Comparative Study between COVID-19 and Bacterial Sepsis Patients

**DOI:** 10.3390/jcm13185603

**Published:** 2024-09-21

**Authors:** Dimitrios-Dorotheos Papadakis, Marianna Politou, Theodoros Pittaras, Ioanna E. Stergiou, Antonia Koutsoukou, Maria Kompoti, Ioannis Vasileiadis

**Affiliations:** 1 Department of Pathophysiology, School of Medicine, National and Kapodistrian University of Athens, 115 27 Athens, Greece; dimitriosdorotheospapadakis@gmail.com (D.-D.P.); stergiouioa@med.uoa.gr (I.E.S.); 2 Haematology Laboratory-Blood Bank, Aretaieion Hospital, National and Kapodistrian University of Athens, 115 28 Athens, Greece; mariannapolitou@gmai.com (M.P.); tpittaras@med.uoa.gr (T.P.); 3 Intensive Care Unit, First Department of Respiratory Medicine, Sotiria Hospital, National and Kapodistrian University of Athens, 115 27 Athens, Greece; koutsoukou@yahoo.gr; 4 Thriassio General Hospital of Eleusis, 190 18 Eleusis, Greece; mariakompoti@gmail.com; 5 1st Critical Care Department, Evangelismos Hospital, National and Kapodistrian University of Athens, 115 21 Athens, Greece

**Keywords:** complement, coagulation, thromboinflammation, COVID-19, sepsis

## Abstract

**Background/Objectives**: Through the past several years, a constant interaction has been implicated between complement and coagulation cascades. SARS-CoV-2 infection and bacterial sepsis are potent activators of both cascades. This study aims to compare the extent of complement and intrinsic coagulation pathway activation (and the interplay between them) among patients with COVID-19 and bacterial sepsis. **Methods**: Serum and plasma samples were collected from 25 ICU patients (11 patients with COVID-19 and 14 patients with bacterial sepsis) at two time points (on admission and either on improvement or deterioration). The activities of coagulation factors XI and XII and complement factors C3a and C5a were measured at both time points. **Results**: The activities of factors XI and XII were increased in both groups of patients and at both time points. However, there were no statistically significant differences between SARS-CoV-2 and bacterial sepsis patients. On the other hand, both C3a and C5a were significantly higher in the COVID-19 group on admission. This correlation was preserved on reassessment. **Conclusions**: Complement activation seems to be more enhanced in COVID-19 than bacterial sepsis. However, the lack of statistical significance in factors XI and XII indicates t the presence of additional pathways for complement activation in SARS-CoV-2 infection.

## 1. Introduction

**Figure 1:**
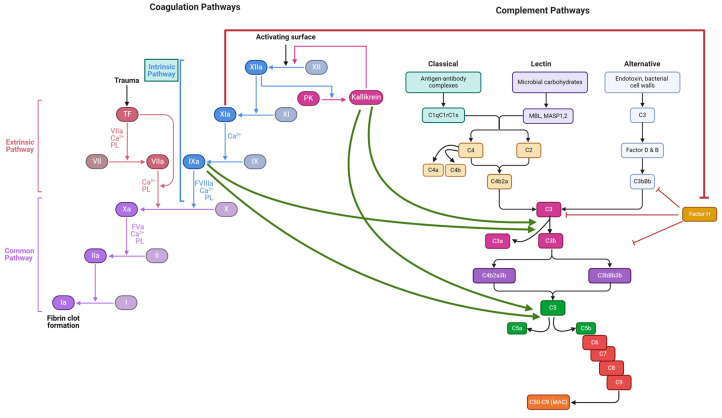
The intrinsic coagulation pathway and the complement cascade interactions. FXIIa enhances the transformation of prekallikrein (PK) to kallikrein. Kallikrein has been shown to induce the release of the anaphylatoxin C5a and the generation of active C3 fragments. Factor FIXa can cleave C3 and C5, generating functional anaphylatoxins C3a, C5a, and C3b or iC3b-like fragments. FXIa has been found to neutralize complement factor H, a major inhibitor of the complement alternative pathway. Sharp arrows indicate activation, while blunt arrows indicate inhibition; created with Biorender.com.

While for many decades, the complement and coagulation cascades were regarded as separate pathways, recently, it has been proven that there is a consecutive interplay between them [1]. Complement proteins can activate the coagulation cascade, while coagulation factors, i.e., thrombin and factor Xa, can directly activate components of the complement cascade. Moreover, the intrinsic coagulation pathway (contact activation or kallikrein/kinin system) lies at the interface between coagulation, fibrinolysis, and complement activation [2]. Activated factor XI (FXIa) and activated factor XII (FXIIa) actuate the complement cascade through different mechanisms; for instance, FXIIa leads to the bradykinin generation and formation of C3a and C5a via the classical complement pathway, while FXIa has been found to neutralize complement factor H, a major inhibitor of the alternative pathway [2]. Figure 1 illustrates the interactions between the intrinsic coagulation pathway and the complement cascade.

Since the pandemic caused by the novel coronavirus disease (COVID-19) broke out, there has been a progressive expansion of knowledge about its underlying pathophysiology. Among other features, endothelial dysfunction [3], coagulation abnormalities [4], and the formation of neutrophil extracellular traps (NETs) [5] have all been found to contribute to the spectrum of disease caused by SARS-CoV-2. Complement hyperactivation has also been implicated in COVID-19-associated thromboinflammation [6].

Likewise, the role of bacterial sepsis in thromboinflammation has been established [7]. Bacterial sepsis also seems to be a potent activator of both the complement and the intrinsic coagulation pathway and thus an inductor of the interaction among them [8].

The purpose of this study is to investigate the potency of the complement and the intrinsic coagulation pathway interaction in each type of infection.

## 2. Materials and Methods

### 2.1. Study Population

This prospective, observational study was carried out in a 10-bed adult Intensive Care Unit (ICU) for 6 months, between May and November 2020. The study was conducted according to the guidelines of the Declaration of Helsinki and approved by the Institutional Ethics Committee of Sotiria Hospital, Athens, Greece. Patients themselves or their next of kin provided informed consent. Patients with bacterial sepsis or SARS-CoV-2 infection who met the sepsis criteria [9] at the time of ICU admission were consecutively enrolled in the study. As this study was performed in 2020 (the first year of the pandemic), no patient was vaccinated for SARS-CoV-2.

### 2.2. Serum Sampling

Blood samples were obtained on admission (time point 1) from every patient and analyzed for routine laboratory parameters. Serum and plasma were stored at −80 °C to estimate coagulation factor XI (FXI) activity, coagulation factor XII (FXII) activity, and C3a and C5a values. Acute Physiology and Chronic Health Evaluation (APACHE) II [10] and Sequential Organ Failure Assessment (SOFA) [11] scores were calculated. The same parameters were reassessed when a significant change in the patient’s clinical condition was noted, either remission or prominent deterioration (time point 2), as defined in another study that was conducted in the same department [12]. Specifically, the remission of sepsis was defined as (a) improvement in systemic inflammatory response syndrome (SIRS) indices (with special emphasis on temperature (T, 36 °C ≤ T ≤ 38 °C) and white blood cell count (WBC, 4000/μL ≤ WBC ≤ 12,000/μL and ≤10% immature forms)); (b) hemodynamic stability for at least 24 hours (systolic blood pressure ≥ 90 mm Hg and mean blood pressure ≥ 65 mm Hg), with no need for vasoactive agents, and lactate concentration <2 mEq/L; and (c) recovery of organ failures attributable to sepsis, with the recovery of organ dysfunction variables and a reduction in SOFA score ≥ 2 compared to the SOFA score at time point 1. Although the SIRS criteria are no longer included in the current definition of sepsis, these markers were employed as they are commonly used by ICU physicians to clinically assess the progress of sepsis. If a patient was discharged from the ICU because they were considered cured by the ICU physicians, we recorded the parameter values on the day of discharge from the ICU. In addition, deterioration of sepsis was defined as (a) worsening hemodynamic instability, with a minimum score of three points in the hemodynamic category, as defined by the SOFA severity scale, and an increase in lactate levels >1 mEq/L compared to time point 1, up to a concentration of ≥2 mEq/L, or increased need for vasoactive substances (norepinephrine increase > 0.1 μg/kg/min, addition of epinephrine or vasopressin), excluding hemorrhagic shock, cardiogenic shock due to an acute cardiac episode or obstructive shock, and (b) the deterioration of organ failure due to the septic syndrome, with a subsequent SOFA score ≥ 1 increase compared to the SOFA score at time point 1 [12].

### 2.3. Diagnosis of SARS-CoV-2 and Source of Bacterial Sepsis 

The diagnosis of SARS-CoV-2 infection was made at the Hellenic Pasteur Institute by applying in-house real-time polymerase chain reaction (RT-PCR) on bronchial secretions. For bacterial infections, blood, urine, and bronchial secretion cultures were utilized. 

### 2.4. Factor XI and XII Activity

FXI and FXII activity was determined by using coagulation FXI deficient plasma and coagulation FXII deficient plasma, respectively. The samples were analyzed by a Siemens coagulation analyzer BCS/BCS XP System (Siemens Medical Solutions USA, Inc., Malvern, PA, USA), employing the chromogenic method. As the coagulation factors’ activities of our patients are expressed as percentage values relative to the normal ones, a reference curve should be generated by employing plasma from at least ten healthy individuals. Normal activities were determined at 70–120% of the reference value for FXI and 70–150% for FXII.

### 2.5. C3a and C5a Levels

C3a and C5a levels were calculated by employing the standard ELIZA (Enzyme-linked immunosorbent assay) method. Human C3a ELIZA Kit and Human C5a ELIZA Kit by Thermo Fisher Scientific (Waltham, MA, USA) were used.

### 2.6. Statistical Analysis

Categorical variables were analyzed with Fisher’s exact test. The normality of continuous variables was assessed with Kolmogorov–Smirnov test. Normally distributed data were analyzed with Student’s *t*-test and one-way ANOVA as appropriate. Skewed data were analyzed with nonparametric methods (Mann–Whitney test or Kruskal–Wallis test as appropriate). Since we had repeated measurements at two time points (first on ICU admission and second upon outcome), we implemented generalized estimating equations (GEEs), an extension of the generalized linear model that accounts for the within-subject correlation. GEEs were used to model the association of various response variables (C3a, C5a, FXI, FXII, etc.) with explanatory variables (particularly type of infection, but also other variables such as sex, age, disease severity scores, etc.). In all GEE models, an unstructured correlation structure was used and the Quasi-Likelihood Information Criterion (QIC) was used for model selection. Data analysis was performed with SPSS 17.0 (IBM Corporation, Armonk, NY, USA, 2008). For all analyses, alpha was set at 0.05 (two-tailed). 

## 3. Results

In total, the data of 25 patients consecutively admitted to the ICU, 18 male and 7 female, were analyzed. The mean age (±SD) was 71.3 ± 11.5 years. All patients were intubated and mechanically ventilated upon their ICU admission. Septic patients with respiratory infection (most of the cohort) had a PaO_2_/FiO_2_ ratio of less than 150. Notably, 3 out of the 11 patients with COVID-19 also had bacterial superinfection on admission. At re-evaluation, none of the improving patients suffered from an active bacterial infection (one patient had pseudomembranous colitis in remission). Three out of four of the deceased patients suffered from an active bacterial infection at re-evaluation.

The mean APACHE II score at admission was 13.4 ±6.5. The mean SOFA score at admission was 7.9 ± 2.1. The mean ICU LOS was 16.7 ± 14.5 days. Crude mortality was 32% (four COVID-19 patients and four patients with bacterial sepsis). Eleven patients had SARS-CoV-2 infection, and the rest had bacterial infection. As previously mentioned, none of the patients were vaccinated against SARS-CoV-2. Overall, 11 of the 14 patients in the bacterial sepsis group had respiratory tract infections, 1 had cholangitis, 1 had MSSA bacteremia, and 1 had sepsis related to large bowel obstruction. Table 1 summarizes the clinical characteristics and laboratory findings of the studied cohort on admission and either on improvement or deterioration.

FXI and FXII activities were increased in both groups, compared to the normal range; however, there was no statistically significant difference between the groups (COVID-19 vs. bacterial sepsis):

fXI1:179.4 ± 37.1 vs. 195.7 ± 25.0%, *p* > 0.05, fXI2: 185.8 ± 33.9 vs. 172.2 ± 61.9%, *p* > 0.05, fXII1: 177.1 ± 47.2 vs. 188.2 ± 42.7%, *p* > 0.05, fXII2: 178.5 ± 48.4 vs. 155.3 ± 80.0%, *p* > 0.05. 

On the other hand, C3a and C5a levels on admission were significantly higher in the COVID-19 group:

C3a1: 26,602.7 ± 23,224.6 vs. 9735.4 ± 7447.1 ng/mL, *p*: 0.018, C5a1:71.1 ± 38.3 vs. 17.2 ± 14.5 ng/mL, *p* < 0.001. 

The correlation was also preserved at patient improvement/deterioration:

C3a2: 32,970.5 ± 18,156.3 vs. 13,216.5 ± 12,006.5 ng/m:, *p*: 0.001, C5a2: 84.5 ± 64.4 vs. 52.0 ± 81.7 ng/mL, *p*: 0.009.

**Figure 2:**
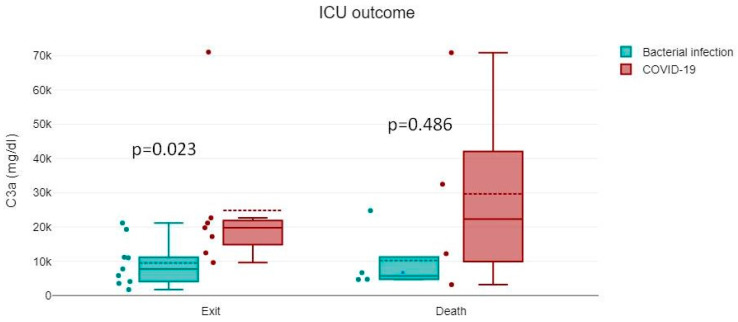

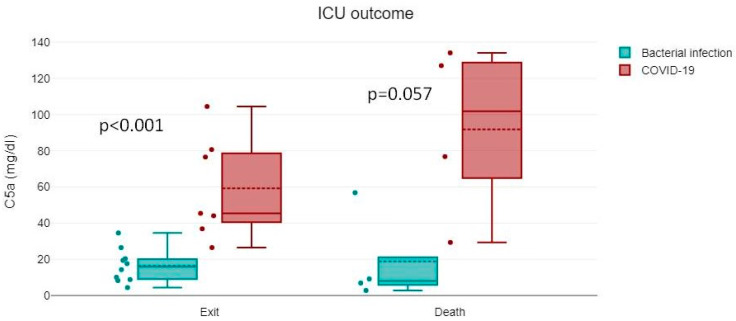
C3a and C5a levels in patients with bacterial infection or COVID-19 with respect to the outcome.

The above-studied parameters showed no statistically significant differences regarding the outcome (i.e., survivors vs. non-survivors, both on admission and re-evaluation) (Figure 2).

Linear regression models (Table 2) give insight into the trends of C3a and C5a levels. Estimates in model 1 indicate that C3a was significantly elevated in COVID-19 patients compared to patients with bacterial sepsis. The mean C3a levels were higher in COVID-19 patients by 12,236.6 ng/ml (95% 4971.9–19,501.3) compared to patients with bacterial sepsis (*p* = 0.01), adjusted for age, sex, and APACHE II. An important finding of our study is that the platelet (PLT) count was independently associated with C3a, i.e., C3a increased by 63.6 ng/m; per 1000/cc increase in the PLT count (*p* = 0.002). Estimates in model 2 indicate that C5a was elevated in COVID-19 patients compared to patients with bacterial sepsis, an association of borderline significance (*p* = 0.047). The mean C5a levels were higher in COVID-19 patients by 30.5 ng/ml (95% 0.4–60.7) compared to patients with bacterial sepsis (*p* = 0.047), adjusted for age, sex, and APACHE II. The PLT count was independently associated with C5a, i.e., C5a increased by 0.14 ng/mL per 1000/cc increase in the PLT count (*p* = 0.011). 

## 4. Discussion

The above results are in accordance with a previous study performed in a different patient population of the same department, demonstrating that while immune stimulation (evaluated by myeloperoxidase) was significantly greater in COVID-19 patients compared to patients with H1N1 or bacterial sepsis, no difference was observed in coagulation factor levels between the different septic groups [13]. The increased levels observed in all the compared parameters can be attributed to the severe inflammation characterizing both bacterial and COVID-19 septic episodes. Bacterial sepsis can induce complement activation by all three pathways [14]. However, evidence suggests different types of bacteria might preferentially induce a specific complement pathway. An interesting example is the significant consumption of C1q (a classical pathway mediator) but not of mannose-binding lectin (the essential mediator of the lectin pathway) in sepsis associated with Gram-positive bacteremia. The reverse finding (increased binding of mannose-binding lectin, preferentially to Gram-negative lipopolysaccharide) was observed in bloodstream infections due to Gram-negative organisms [15]. SARS-CoV-2 infection has also been found to induce all three complement pathways [6,16]. The SARS-CoV-2 envelope proteins bind to mannose-binding lectin, activating the lectin pathway. Also, SARS-CoV-2-specific antibodies induce the classical pathway via C1q. Finally, SARS-CoV-2 triggers the alternative pathway by suppressing the inhibitory effects of complement factor H on C3. Complement inhibition possibly favors clinical remission from severe COVID-19 infection. C5a blockade by eculizumab, a monoclonal antibody used primarily to treat paroxysmal nocturnal hematuria (PNH), showed more than encouraging results in multiple reports when used for critically ill patients suffering from COVID-19 [17,18,19].

A proposed explanation for the disproportionately higher C3a and C5a levels in COVID-19 patients compared to the septic ones might be the simultaneous operation of all complement pathways in COVID-19. On the contrary, as previously mentioned, a specific complement pathway seems to predominate in bacterial sepsis, and this is probably related to the individual bacterial agent.

The concept of complement activation in COVID-19 seems to gain renewed interest as it has been implicated as an important player in long COVID [20]. It should also be noted that the rate of complement activation and coagulopathy in COVID-19 may differ among different SARS-CoV-2 variants [21].

FXII and FXI activities were found to be elevated in both COVID-19 and bacterial sepsis patients but without statistically significant differences between the groups. The contact activation system (CAS), the most upstream component of the intrinsic coagulation cascade, consists of coagulation factors XI (FXI) and XII (FXII), prekallikrein (PK), and high-molecular-weight kininogen (HK). When activated, CAS promotes the recruitment of leukocytes and PLTs, the initiation of the complement cascade, and the generation of bradykinin, in addition to its more recognized role, which is the initiation of coagulation [22]. 

An increasing number of factors seem to be potent CAS initiators, including infectious pathogens and cell-free DNA. In sepsis, bacterial cell components, such as peptidoglycans, teichoic acid, lipopolysaccharide, and PLT-derived polyphosphates, lead to CAS initiation. FXII is activated, thus becoming the active serine protease FXIIa. FXIIa leads to the formation of kallikrein (PKa) by the activation of prokallikrein (PK). FXIIa activates FXI to FXIa, which leads to the initiation of the intrinsic coagulation pathway [22]. As mentioned at the beginning of the present study, the intrinsic coagulation pathways FXI and FXII are per se potent activators of the complement system [1,2] via distinct mechanisms. According to a previous study, the activated form of FXI was increased in septic patients, with a correlation between the levels of FXIa and illness severity (evaluated by the APACHE score and PLT count) [23]. No relevant results were observed in the present study, possibly due to the lower number of patients with bacterial sepsis (14 vs. 32).

The significance of FXI and FXII in the pathobiology of sepsis has been highlighted in several studies in which they were evaluated as possible targets of therapy. In a baboon model of *Staphylococcus aureus*-induced sepsis, FXII-neutralizing antibodies prevented the uncontrolled activation of FXI, kallikrein, and kininogen. As a result, a milder immune response was observed in the antibody-receiving animals compared to the non-antibody-receiving ones [24]. A relative decrease in plasma biomarkers of inflammation, such as TNF, interleukin (IL) 1-β, IL-6, IL-8, IL-10, and myeloperoxidase, was also observed [24]. In a murine study of bacterial pneumonia either by *Klebsiella pneumoniae* or *Streptococcus pneumoniae*, FXII deficiency was found to provide a survival benefit to the mice infected by *K. pneumoniae*. However, FXII deficiency did not affect the survival of the mice infected by *S. pneumoniae* [25]. FXII inhibition has also been proposed as a protective approach against severe infections. In a murine model of polymicrobial sepsis, the exogenous administration of an anticoagulant antibody that selectively blocks FXI activation by FXII led to reduced IL-6 and TNF-a, reduced PLT consumption, and improved overall survival [26]. In a subsequent study, in which the same antibody was administered in baboons infected with *S. aureus*, FXI inhibition was found to be protective against sepsis-induced organ failure and disseminated intravascular coagulation, also leading to reduced mortality [27]. Further experimental studies are also in accordance with the aforementioned results [28,29,30].

However, some data emphasize the prophylactic effect of the intrinsic coagulation pathway on infection. In a previously mentioned study, FXI knockout mice with bacterial pneumonia either by *K. pneumoniae* or by *S. pneumoniae* increased mortality compared to controls [22]. A probable interpretation might be given based on the extent of the inflammatory process; that is to say, in infections confined to specific organs, FXI may tend to express its protective effects, while in more severe infections where inflammation prevails all over the body, the intrinsic coagulation pathway initiation probably leads to unfavorable outcomes.

An overt stimulation of the intrinsic coagulation pathway has also been established in COVID-19. In a 2022 study, the intrinsic coagulation pathway mediator activities (FXIa and FXIIa among them) were measured in healthy individuals and patients with SARS-CoV-2 infection [31]. The aforementioned mediators were elevated in the COVID-19 group. Moreover, among the COVID-19 patients, higher levels of these mediators were proportionally associated with disease severity [31]. FXII expression and activity were found to be elevated in the lung parenchyma, within the pulmonary vasculature, and the fibrin-rich alveolar spaces of post-mortem lung tissues extracted by recently deceased COVID-19 patients [32]. This was attributed to NET accumulation, which leads to FXII activation [32]. However, the administration of garadacimab, a monoclonal antibody against FXII, failed to show any benefits regarding survival [33]. On the other hand, there is a paucity of data on the role of FXI in COVID-19-related thromboinflammation.

Another highlight of our study was that an increase in the PLT count was observed in parallel with the levels of C3a and secondarily with C5a levels. Notably, previous studies have identified the role of PLTs as a potent activator of the complement system and inflammation in general [34]. Chondroitin sulfate, a glycosaminoglycan released by PLT alpha granules, acts as a potent initiator of complement activation. As a consequence, PLT activation leads to the generation of C3a and soluble C5-9 complexes. At the other end of the spectrum, C3a has been found to exert stimulatory effects on PLTs through the C3a receptor [21]. 

The main limitation of our study is the low number of patients. Therefore, conclusions cannot be drawn about possible differences between patients in remission and those undergoing deterioration. Nevertheless, a recent study that assessed the possible correlation between the concentrations of several complement factors and sepsis severity found that there was no relationship between the levels of complement factors (C3a and C5a among them) and the outcome. The proposed explanation is that in sepsis and septic shock, the complement system is already profoundly activated and is no longer related to the clinical outcome [35]. This study’s main advantage is the comparison made between COVID-19 patients and septic patients suffering from bacterial infection. To our knowledge, there is no comparative study that highlights the disparity in complement activation between bacterial sepsis and COVID-19. Regarding other chronic conditions (i.e., cardiovascular, respiratory, and renal), in which the complement and coagulation cascade activation is either a driver or a result of the disease pathophysiology, no statistically significant differences in the coexistence of chronic disease were observed between COVID-19 and bacterial sepsis patients. We should also note that previous studies have shown that young adults with COVID-19 hospitalized in the ICU had a sepsis rate of 65.5%, with pulmonary bacterial coinfection being the most frequent (59.2%) [36]. In our cohort, besides the fact that about 27% of COVID-19 patients (i.e., 3 of the 11) presented with bacterial superinfection on admission, we should note that the overwhelming complement activation in COVID-19 patients compared to patients with bacterial sepsis suggests that SARS-CoV-2 infection is the predominant factor for the increase in C3a and C5a. It should be emphasized that, as this study was performed in 2020, none of the patients with SARS-CoV-2 infection were vaccinated. Consequently, the clinical course of our COVID-19 patients probably resembles the natural course of the disease. Nevertheless, as new SARS-CoV-2 variants continue to emerge, conclusions cannot be safely drawn about the natural disease course caused by the newer variants. Also, despite the low number of patients, the main findings of our study (excessive C3a and C5a activation in COVID-19 patients) are consistent in every applied statistical model. Because of the small sample size (n = 25), all the initial comparisons were performed with the use of nonparametric tests, which require no assumptions of normality but are more conservative in depicting statistical significance. Furthermore, in model fitting, we used generalized estimating equations (GEEs), which do not require assumptions of normality for either predictor or response variables or for model residuals. However, a larger sample would allow for more robust estimates.

## 5. Conclusions

Our study demonstrated considerable complement and coagulation system activation in both the COVID-19 and bacterial sepsis patient groups, with a more profound complement cascade enhancement in SARS-CoV-2 infection. Furthermore, proportional relationships between the PLT count and both C3a and C5a were established. However, further studies are required to elucidate the differences in the pathophysiology of these two entities.

## Figures and Tables

**Table 1:** Demographics as well as clinical and laboratory parameters among COVID-19 and bacterial sepsis patients.

Variable	COVID-19 (n = 11)	Bacterial Sepsis (n = 14)	*p* Value
Age (years)	71.8 ± 10.2	70.9 ± 12.8	0.936
Male sex	6 (54.6%)	12 (85.7%)	0.039
Chronic cardiovascular disease	4 (36.4%)	5 (35.7%)	0.487
Chronic respiratory disease	3 (27.3%)	6 (42.9%)	0.204
Chronic renal disease	1 (9.1%)	1 (7.1%)	0.570
APACHE II score at ICU admission	12.7 ± 6.1	13.9 ± 6.9	0.727
SOFA score at ICU admission	7.4 ± 1.4	8.3 ± 2.5	0.291
FXI1 (70–120%) on admission	179.4 ± 37.1	195.7 ± 25.0	0.314
FXI2 (70–120%) on improvement/deterioration	185.8 ± 33.9	172.2 ± 61.9	0.893
FXII1 (70–150%) on admission	177.1 ± 47.2	188.2 ± 42.7	0.687
FXII2 (70–150%) on improvement/deterioration	178.5 ± 48.4	155.3 ± 80.0	0.727
C3a1 (50–200 ng/mL) on admission	26,602.7 ± 23224.6	9735.4 ± 7447.1	0.018
C3a2 (50–200 ng/mL) on improvement/deterioration	32,970.5 ± 18156.3	13,216.5 ± 12,006.5	0.001
C5a1 (14.7–30.2 ng/mL) on admission	71.1 ± 38.3	17.2 ± 14.5	<0.001
C5a2 (14.7 30.2 ng/mL) on improvement/deterioration	84.5 ± 64.4	52.0 ± 81.7	0.009
PLT1on admission	217,270.0 ± 144,567.8	184,064.3 ± 114,467.3	0.344
PLT2 on improvement/deterioration	296,845.5 ± 149,004.2	238,007.1 ± 141,582.2	0.244
Death in the ICU	4 (36.4)	4 (28.6)	0.679

**Table 2:** Linear regression models for C3a and C5a.

Variable	Beta Coefficient	95% Confidence Interval	*p* Value
**Model 1: C3a** (adjusted for age, sex, and APACHE II)			
Infection type			
COVID-19	12,236.6	4971.9–19,501.3	0.001
Bacterial (ref)	0		
PLT (per 1000/cc increase)	63.6	24.3–103.0	0.002
**Model 2: C5a** (adjusted for age, sex, and APACHE II)			
Infection type			
COVID-19	30.5	0.4–60.7	0.047
Bacterial (ref)	0		
PLT (per 1000/cc increase)	0.14	0.03–0.24	0.011

## Data Availability

The datasets generalized or analyzed in this study are not publicly available. Researchers wishing to further analyze these datasets must communicate with the corresponding author (I.V.) or D.D.P via their emails.
